# Development, feasibility and acceptability of a self-efficacy-enhancing smartphone application among pregnant women with gestational diabetes mellitus: single- arm pilot clinical trial

**DOI:** 10.1186/s12884-022-04684-1

**Published:** 2022-04-23

**Authors:** Iman Al Hashmi, Hilal Alsabti, Omar Al Omari, Yusra Al Nasseri, Atika Khalaf

**Affiliations:** 1 College of Nursing, Sultan Qaboos University, Muscat, Oman; 2 Sultan Qaboos University Hospital, Sultan Qaboos University, Muscat, Oman; 3 School of Nursing, Midwifery and Paramedicine, Curtin University, Perth, WA Australia; 4 Oman College of Health Sciences, Nursing Program, Ministry of Health, Muscat, Oman; 5 Faculty of Health Sciences, Kristianstad University, Kristianstad, Sweden

**Keywords:** Diabetes, Intervention research, Health behaviors, Pregnancy, Information technology, Self-efficacy

## Abstract

**Background:**

There is growing attention to the use of mHealth technologies to promote glycemic control for women with GDM around the world, but research on promoting a change in health behaviors is lacking. This study aimed to document the process of designing, developing, and testing the feasibility and acceptability of the SEESPA.

**Methods:**

This single-arm pilot clinical trial study included 15 pregnant women with GDM. Following SEESPA development (e.g., goal setting and action plan, role modeling, motivational messages, mastery of experiences, and tracking healthy behaviors), all participants were provided access to use the SEESPA for 4 weeks. Feasibility outcomes assessed were rates of recruitment, retention rate, success rate of transmitting motivational text messages, rate of participants acknowledging receipt of text messages, and success rate of recording healthy behaviors. Acceptability outcomes were determined by asking open-ended questions through telephone interview at 4-week post-intervention.

**Results:**

Fifteen randomly selected women consented to participate in the study, with a 60.0% (*n* = 9) retention rate at post-trial intervention and 40.0% (*n* = 6) trial dropout. Two motivational text messages per week were sent to all participants. Of these, 68.1% were acknowledged by the participants. Study participants reported that SEESPA is useful, effective, and they felt satisfied about it. In addition, they brought few suggestions that will be integrated on the final version of the app.

**Conclusions:**

and Clinical Relevance.

The developed innovative SEESPA is a feasible and acceptable intervention for behavioral modifications among women with GDM, and is ready to be tested in a larger RCT study which is expected to inform the health policymakers to integrate SEESPA with the antenatal health care practice of women with GDM, specifically in developing countries where there is a greater risk of developing GDM complications among mothers and their infants.

Trial registration.

The study is registered on September 16, 2019 (ACTRN12619001278123p) by the Australian New Zealand Clinical Trials Registry.

**Supplementary Information:**

The online version contains supplementary material available at 10.1186/s12884-022-04684-1.

## Introduction

Gestational diabetes is the leading metabolic health condition during pregnancy that consequently contributes to the prevalence of diabetes and obesity across generations. For instance, pregnant women with gestational diabetes mellitus (GDM) are at a sevenfold higher risk to develop type 2 diabetes within 5 to 10 years postpartum (Bellamy, Casas, Hingorani, & Williams, [4]). Further, fetal intrauterine exposure to prolonged hyperglycemia has been found to double the risk for childhood obesity and development of type 1 diabetes (Gilbert et al., [12]).

Most often, developing gestational diabetes requires the pregnant woman to modify her lifestyle behaviors (i.e., diet and physical activity) to control the elevated blood glucose level. Evidence supports the effectiveness of self-care measures (e.g., controlling body weight, healthy diet, physical activity, and self-monitoring of blood glucose [SMBG]) to control blood glucose levels and lower the risk for development of overt diabetes, but behavioral changes could be challenging, in particular during a short span of pregnancy (Carolan, Steele, & Margetts, [7]). The World Health Organization ‘Making Pregnancy Safer’ initiative, affirms that optimizing maternal health by supporting women’s active role and enhancing their capacities for making healthy choices is a fundamental nursing role (World Health Organization, [30]). Therefore, prenatal nurses should consider integrating motivational strategies in any prenatal health promoting interventions, beyond those traditional health education interventions that limit mothers to being passive recipients of health information. 

Women diagnosed with GDM represent an exceptional group of the population to examine innovative motivational strategies to deliver behavioral-based health promoting interventions. This is because GDM is a temporary health problem diagnosed in the third trimester of the pregnancy, and it usually reverts to normal during the postpartum period (Plows et al., [25]). Therefore, those pregnant women are not always entirely informed about gestational diabetes and its impact on pregnancy outcomes. Also, women with GDM face challenging demands of housework, child care, family, and work commitments that make it difficult for them to change their behaviors within a short period of time. Therefore, developing innovative health promoting interventions that is adapted to specific gaps in knowledge and motivation of these women is crucial to enhance their active role by undertaking the recommended healthy behaviors for glycemic control.

Nowadays, the use of mobile health (mHealth) technologies is widely used as a source of health information for pregnant women (Nwolise, Carey, & Shawe, [23]; Borgen et al., [5]). Pregnant women are mostly from the younger generations who are familiar and interested in using technological devices such as smartphone apps. Many of them utilize mHealth as a primary source to increase their awareness about pregnancy and as a way to deal with concerns related to pregnancy (Garnweidner-Holme et al., [10]; Pellegrini, Pfammatter, Conroy, & Spring, [24]). Although the previous smartphone app based clinical trials showed promising findings for SMBG among women with GDM; none of them used motivating strategies to promote behavior change and none of them included comprehensive tracking of self-care measures (e.g., body weight, diet, physical activity, and SMBG). Further, none of the previously tested smartphone apps for women with GDM were in Arabic language or were culturally-sensitive to Arabic-speaking pregnant women with GDM.

Since the prevalence of GDM is higher among certain ethnic groups of women from West Asia and northern Africa (ie., dominated with Arabic-speaking people) (Gasim, [11]; Macaulay, Dunger, & Norris, [18]), health information on GDM needs to be easily understandable, motivating, accessible, and culturally tailored to meet women’s needs. Self-efficacy-enhancing interventions has been found to be highly effective among pregnant women with GDM (Al-Hashmi et al.,[1]). In the current study, we proposed a novel self-efficacy-enhancing smartphone application (SEESPA). The main purpose of this study was to examine the feasibility and the acceptability of a newly developed smartphone app, which is the SEESPA, among pregnant women with GDM.

## Methods

### Theoretical framework

The health belief model (HBM) guides the development of SEESPA. The main tenet of the HBM theoretical framework is grounded on the subjective belief of the individual rather than the individual’s experience (Thomas, [28]). The model posits one’s beliefs may serve as motivators that enhance readiness or as barriers that hamper readiness to undertake healthy behaviors (Thomas, [28]). There are six concepts recognized in the HBM as strong and influential factors that determine the individual’s decision whether to modify lifestyle behaviors (Rosenstock, Strecher, & Becker, [26]). These concepts include perceived susceptibility to disease, perceived severity of disease, perceived benefits of health behavior, perceived barriers to health behaviors, cues to action, and the later added concept, self-efficacy (Rosenstock, Strecher, & Becker, [26]). These factors were considered during the development of SEESPA with more focus on using different strategies to enhance self-efficacy for adherence to healthy behaviors.

### Study design and setting

This single-arm pilot clinical trial was conducted between May and June 2021 on the antenatal care clinic at Sultan Qaboos University Hospital (SQUH), Muscat, Oman. The study ethical approval was obtained from the Medical Research Ethical Committee at SQUH. The standard antenatal care for pregnant women with GDM in the study setting includes routine antenatal visits, monthly blood sugar profiles, fasting blood sugar test every visit, SMBG at least twice per week at home, and a one-to-one individualized education visit with a diabetes dietician.

### Sample

**Fig. 1:**
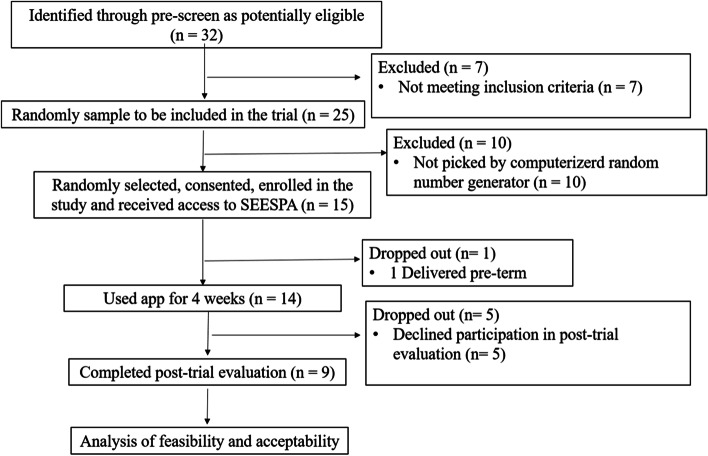
Study Flow Diagram. Abbreviation: SEESPA, self-efficacy enhancing smartphone application

As this is a feasibility pilot study, power calculation for sample size determination is not required (Arain, Campbell, Cooper, & Lancaster, [3]). Random sampling of the clinic roster list of 25 pregnant women with GDM was conducted using a computerized random number generator (Fig. 1). Fifteen Arabic-speaking pregnant women with GDM were recruited from the antenatal clinic at SQUH in Oman. The inclusion criteria included Arabic-speaking women, ≥ 18 years old, with a single pregnancy, between 22–34 weeks of gestation, diagnosed with GDM, attending the study settings during the study timeframe, and able to speak, read, and write in Arabic language. Exclusion criteria included women with multiple gestations, those diagnosed with type 1 or type 2 diabetes, have chronic medical problems, or mental illness, and those who have pregnancy complications that require complete bed rest.

### Procedures

A two-phase process was performed to develop and test the feasibility and acceptability of SEESPA.

### Phase 1: Development of the SEESPA

**Fig. 2:**
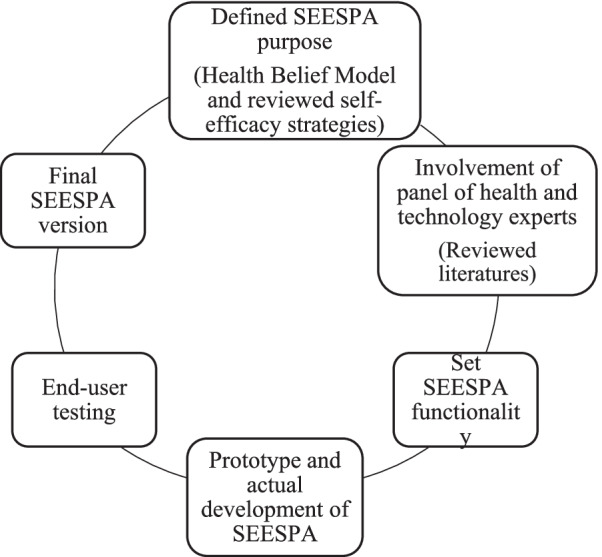
Development Process of SEESPA Abbreviation: SEESPA, self-efficacy enhancing smartphone application

SEESPA was developed to promote self-efficacy of women with GDM and their ability to modify their lifestyle behaviors through the integration of evidence-based self-efficacy enhancing strategies (e.g., goal setting and action plan, role modeling, motivational messages, and mastery of experiences) (Al-Hashmi et al., [1]). This smartphone app was designed to encourage change of lifestyle behaviors and promote adherence to self-care activities such as weight control, SMBG, following a healthy diet, and regular physical activities. Figure 2 illustrates the development process of the SEESPA.

A panel of 7 volunteer experts, invited from the study site, assisted in designing the SEESPA. These included 1 expert in informatics and technology, 1 diabetes nurse, 1 nurse midwife, and 4 nurse researchers. In the first phase of designing the app, the experts discussed the main themes. Eight core sections were suggested by the experts: [1] patient individualized profile, [2] health education content about GDM, [3] note on goal setting and action plan, [4] recommended physical activities, [5] diet recommendations, [6] track healthy behaviors, [7] role modeling, and [8) motivational messages.

The expert group also discussed and decided the content of the SEESPA after reviewing the available literature to ensure the validity and the quality of the health information included in the app (Boulos et al., [6]). The GDM health education content was adapted from the National Institutes of Diabetes and Digestive and Kidney Diseases (NIDDKD) health information (NIDDK, [21]). This information has been translated previously from English to Arabic by the PI of the current study (Al-Hashmi et al., 2018). The readability of the content is suitable for 8th grade reading level using the Simple Measure of Gobbledygook (SMOG) Index. The content included information related to GDM (Table 1).

**Table 1:** Key Sections of SEESPA

**Section**	**Aspect of the section**		**Purpose**
GDM health education	•This section included the following written information about GDM: 1)General information about definition, etiology, risk factors, and treatment of GDM 2)Maternal and neonatal complications related to GDM 3)Importance of healthy lifestyle behaviors such as weight management, healthy diet, exercises, and SMBG level to prevent GDM complications 4)Strategies to prevent type 2 diabetes post-partum •The textual contents are organized in icons according to the section heading so, the user can click on any icon to read the related information		Promote knowledge about GDM perceived, increase perceived susceptibility to disease, increase perceived severity of disease, and increase perceived benefits of following the recommended healthy behaviors
Diet recommendation		•The diet section includes eleven icons that contain information about: 1)General diet advice for women with GDM 2)Fruits (including sugar level) 3)Vegetables (including sugar level) 4)Grains (include the list of grains with carbohydrate level) 5)Milk and dairy (including saturated fat level) 6)Food rich in protein 7)Healthy high-fat foods 8)Food high in fiber 9)Food high in carbohydrates 10)Healthy high carbohydrates food 11)Food high in calories	To promote knowledge and competence to select the appropriate diet to control the BS level
Physical activity		•The physical activity section includes three icons that contain information about: 1)General advice about physical activity during pregnancy 2)Warning signs to stop exercising 3)Recommended physical activities (include list of photos of safe activities that can be done during pregnancy) •The user can navigate between the three icons to educate herself about physical activity during pregnancy	To promote knowledge and competence to choose the appropriate and safe physical activity during pregnancy to control BS level
Goals		•Tapping the goal icon will direct the user to a list of pre-identified goals (selected goals) from which the user can select as many goals she is willing to achieve during a certain period of time. On the same screen, there is an icon for the achieved goals, so after a certain period, the user can check the achieved goals	To motivate the user to achieve the selected goal by following healthy behaviors
Track		•Taping the track section will direct the user to a screen with five track icons that are: 1)Track weight. Tapping the track weight icon will direct the user to a screen where she can enter a new weight record. On the same screen, information about the date and user’s pre-pregnancy BMI will be shown. On the same screen, the user can click on the icon titled ‘previous records’ where she will be see all weight records per date, total weight gain, minimum allowed total weight gain, and maximum allowed weight gain of the user according to her pre-pregnancy BMI. The user will receive a prompt alert if she exceeds the allowed total weight for her BMI 2)Track BS. Tapping the track BS icon will direct the user to a screen where she can enter BS recordings during fasting, 1-h after meal and 2-h after meal. The frequency of BS recordings depends on the advice received by the user’s primary health care provider. The user will receive a prompt alert if the recorded blood sugar is too high or too low On the same screen also, the user can click on an icon titled “previous records” where she will be able to see the current month calendar, and a graphical illustration of her BS readings 3)Track physical activity. Tapping the track physical activity icon will direct the user to a screen where she can select the type of activity performed per day from a list of activities such as brisk walking, swimming, stretching exercise, etc. Following the selection of the activity, will direct the user to a pop-up screen where she can enter and save the time spent doing a specific activity. The user can select as many activities done per day. On the same screen also, the user can click on an icon titled ‘previous records’ where it will illustrate the date, type of activity, and time spent per activity 4)Track diet. Tapping the track diet icon will direct the user to a screen where she can select the type of food consumed per day from a list of foods such as fruits, vegetables, grains, milk and dairy, sweets, and junk food. Tapping any of the food types, will enable the user to record the food consumed and record the sugar level (low or high), or fat level (low or high), or carbohydrate level (low or high) based on the health information provided in the diet section 5)Change in the treatment plan. Tapping this icon will enable the user to record any change in treatment plan after starting use of the app	To record and keep track of self-care behaviors including weight control, diet, physical activity, and BS
Role model		•Tapping the role model icon will direct the user to a recorded video where the role model woman with previous GDM diagnosis talks about her experience of self-managing GDM, barriers and challenges, and motivating factors	To motivate the user to manage the barriers and challenges using different approaches for an optimal pregnancy outcome
Motivational messages		•Tapping the motivational messages will direct the user to a list of received motivational messages. The user receives two automatically set motivational messages per week which are saved later in this section	To motivate the user to adhere to the recommended healthy behaviors for an optimal pregnancy outcome

Abbreviations: SEESPA, self-efficacy-enhancing smartphone application; GDM, gestational diabetes mellitus; BMI, body mass index; SMBG, self-monitoring of blood glucose; BS, blood sugar

In the second step of designing the SEESPA, the PI continued to discuss with the development team, focusing on software development, technical functions, and incorporation of self-efficacy enhancing strategies. The development team of the SEESPA included 2 professional designers and experts in software development to ensure design of a user-friendly interface and highly automated smartphone app (Boulos et al., [6]). The designers developed initial mock screens illustrating the skeletal framework of the app. These mock screens were used to identify the app layout and the core themes. They also helped the PI understand the app’s flow and provided the first impression of how the app screen folders would look like and how they would work. Thereafter, screens were updated based on the output received from the PI. Upon finalizing the user interface, the actual development of the SEESPA started. The SEESPA prototyping was designed considering the following key features:

1. *Data privacy. *The data entered by the user is private, hence all necessary implementation was made to keep it safe from misuse. Data privacy was achieved through the usage of secure connection for data transactions, usage of complicated security keys for each user, and by protecting access to the administration section through secure login.
2. *Ease-of-use.* As the users are non-technical, the app was designed to be simple, attractive, and easy to use and follow.
3. *Information presentation.* Since the devices have small screens, the health information was efficiently and effectively arranged in a way that the user can easily read and understand.
4. *Patient profile. *The PI can register participants’ information for the first-time on the administration page of the app and create a unique password for each participant to use for log-in.
5. *Track healthy behaviors.* Participants were able to key-in the following healthy behaviors into the app: 1) physical activities, 2) blood sugar levels, 3) dietary history, and 4) body weight. The cultural sensitivity aspects and the practicality of the recommended exercises for pregnant women were considered by the study authors who are Arabic-speaking when preparing the SEESPA. The study authors are aware of and immersed in the participants’ cultures because they share the same cultural background, language preferences and belief system with the study participants. Thus, all these factors were taken into consideration when developing the SEESPA. The self-reported data on body weight, healthy diet, physical activity, and blood sugar monitoring were available for viewing in a graphical form that thus supports participant’s adherence to the recommended healthy behaviors (Table 1).
6. *Self-efficacy enhancing strategies.* Self-efficacy enhancing strategies integrated in the SEESPA include goal setting and action plan, role modeling, motivational messages, and mastery of experiences. A sample motivational Message, “Dear Mom: You can do a lot to prevent or delay type 2 diabetes in your life by making a healthy lifestyle decisions such as staying at a healthy weight, being physically active for at least 30 min most days of the week, and following a healthy eating plan”. As for Mastery of experiences, an educational video demonstration of SMBG level was available in the health education content section for the participants to watch and practice at any time. In the study setting, women with GDM usually receive a glucometer to check their blood glucose level at home after receiving an instruction session on blood sugar monitoring by a staff nurse (Table 1).
7. *Track usage time.* The SEESPA is expected to track the usage time of each section.

**Fig. 3:**
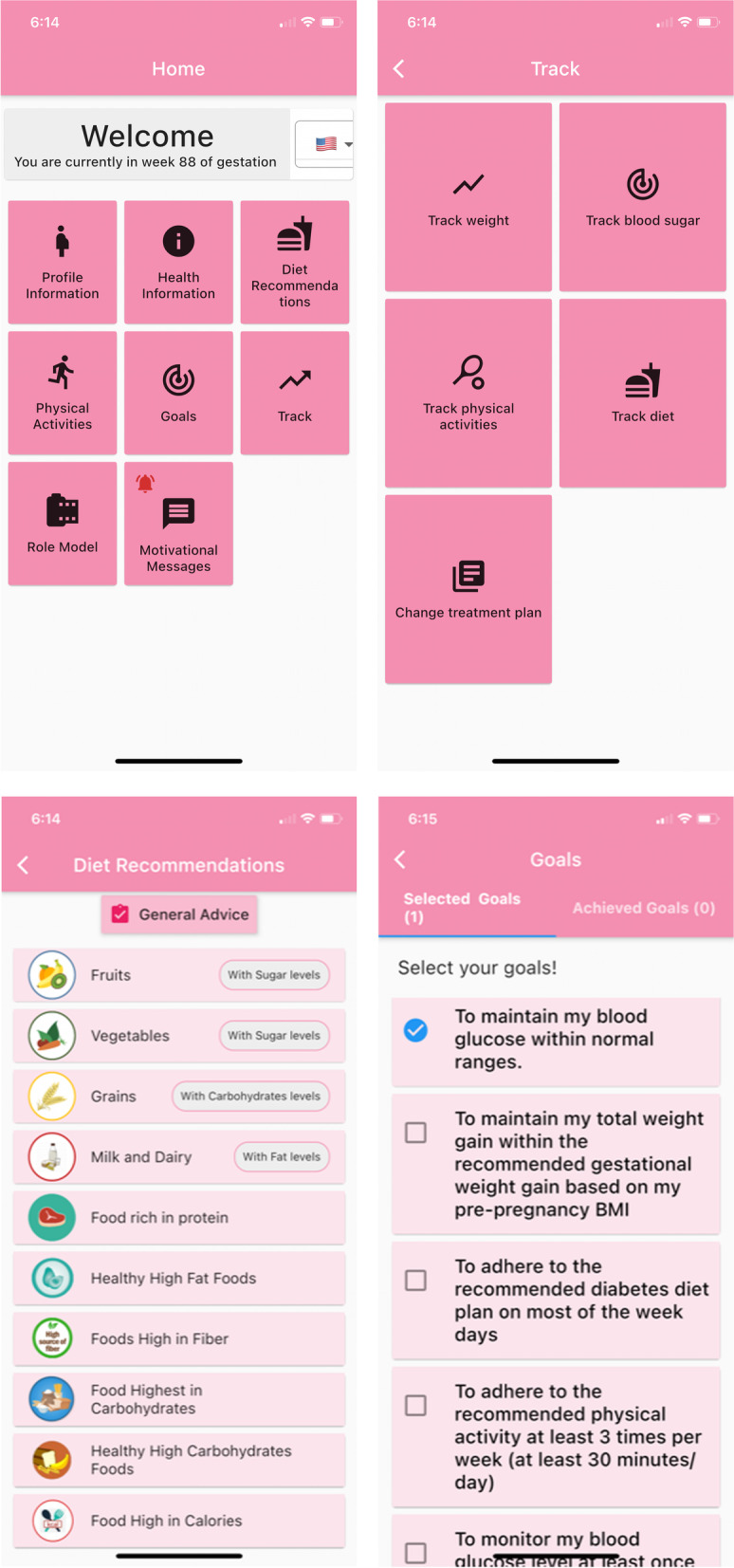
Screenshots of SEESPA

In addition, to address the needs of the Arab-speaking mothers, the SEESPA was developed for smartphones running the most commonly operating systems (iOS and Android). Further, the app was designed in both English and Arabic languages. Figure 3 shows screenshots of the SEESPA. The core aspects of the app features and their purpose were shown in Table 1.

### Phase 2: Procedure for testing SEESPA feasibility and accessibility

The 15 participants consented following protection of human subjects’ approval of the study which was obtained from the Medical Research Ethics Committee at SQUH (Fig. 1). A two-phases screening procedure was implemented to assess participants’ eligibility for pilot-test participation. First, potential participants were asked to complete a self-administered screening form during their antenatal visit to determine their eligibility for participating in the study. Second, the PI invited the eligible participants to enroll in the pilot study. Written informed consent was obtained from all the participants before the start of the study. A 10-min meeting with the PI was followed for instruction on smartphone app operation and data entry steps required for recording participants’ responses. It was anticipated that the participants will utilize the app for 4 weeks to access GDM health-related information, and record and track their blood sugar level, body weight, diet behaviors, and physical activity. Participants were instructed not to share the smartphone app with others. After enrollment in the trial, participants were given a unique password to gain access to the application. The participants were informed that the app was designed for research purposes only and their usage would be monitored until the end of the trial period.

Participants’ baseline data (participant’s code, age, BMI, gestational age, last menstrual period, expected delivery date, and GDM treatment) were retrieved from their medical records by the PI, who later entered this information in the participants’ profile screen through the SEESPA administration page to personalize GDM-related self-care activities of the users. At the end of the pilot study, a thank message was sent to all the participants as an appreciation for the time they spent during the study.

### Outcome measurement

The primary aim of the study was to assess the feasibility of the SEESPA among pregnant women with GDM. The feasibility of the study was determined by rates of recruitment, retention rate in the study, success rate of transmitting motivational text messages, rate of participants acknowledging receipt of text messages, and success rate of recording healthy behaviors (e.g., body weight, blood sugar, food intake, physical activity) on the app. At the end of the trial, data regarding recorded healthy behaviors and received motivational text messages on the smartphone app was downloaded from the administration section of the SEESPA for data analysis. The data collected through the smartphone app was encrypted and secured with a password on the computer.

The secondary aim of the study was to assess the acceptability of SEESPA among women with GDM. The acceptability was determined by conducting individual telephone interview at 4-week post-intervention. A focus group session with the study sample at the end of 4 weeks was planned to determine the acceptability of the app. However, because the data collection happened during the COVID-19 pandemic, meeting the study participants face to face in a group was not possible as per the policy of the study setting. Therefore, individual telephone interviews were conducted to collect the required data. Study participants were asked if it was convenient for them to meet online using ZOOM or Google Meet platforms, but all declined to participate through these platforms and preferred telephone call.

Five open-ended questions were asked to the participants: (1) How was your experience with the trial version of the SEESPA in terms of usefulness, effectiveness, and satisfaction to improve self-efficacy and adherence to healthy behaviors? (2) How many times have you accessed and used the app per week? (3) What are your suggestions to improve the SEESPA? (4) What are the perceived motivating factors for using SEESPA? (5) What are the perceived barriers for using SEESPA? Information obtained from individual telephone interviews aided in improving the final version of SEESPA. Self-efficacy was explained to the participants as their belief about their ability to effectively perform the recommended behavior (Rosenstock, Strecher, & Becker, 1988).

### Data analysis

Quantitative (e.g., recruitment rates, retention rate, success rate of transmitting motivational text messages, and rate of participants acknowledging receipt of text messages) and qualitative data were analyzed manually by the PI. Qualitative data were reported under 6 themes: (1) usefulness of SEESPA, (2) effectiveness of SEESPA on improving self-efficacy, (3) satisfaction with SEESPA, (4) barriers to using SEESPA, (5) motivating factors to use SEESPA, and (6) suggestions for improvement.

### Ethical consideration

Participants’ confidentiality was maintained by allocating code numbers for each participant and the PI was the only person who had access to the master list. All of the participants’ information was locked in a secure cabinet in a locked room. Data entered into the SPSS (IBM Corp. Released 2016. IBM SPSS Statistics for Windows, Version 24.0. Armonk, NY: IBM Corp) program has all identifiers coded to protect the participant’s identity, and the PI has only limited access in this information. Informed consent was obtained from all participants.

## Results

### Feasibility of the SEESPA

A total of 32 women with GDM were screened for eligibility from May to June 2021. Twenty-five of them met the inclusion criteria. Of these, 15 women were randomly selected and were invited to participate in the study. With regard to the recruitment rate, 100% (*n* = 15) of the invited women consented to participate, with 60.0% (*n *= 9) of them continued utilization of the app (i.e., 4 weeks) and agreed to participate in post-trial evaluation (retention rate). Trial dropout was 40.0% (Fig. 1). The reasons given for the dropouts were personal issues (*n* = 5) and preterm delivery (*n *= 1). The majority (*n* = 7, 77.7%) of the participants were using iOS. The participants mean age was 30.6. Two thirds (77.8%) of the study participants had an educational level equal to college or college graduate (Table 2).

**Table 2:** Participants’ Characteristics

Characteristic	Total Sample *n* = 9
Age, years, mean	30.6
Body mass index, mean	23.4
Education level, n (%)	
Less than high school	0
High school graduate	1 (11.1)
College, graduate or under graduate	7 (77.8)
Master’s degree graduate	1 (11.1)
GDM Treatment, n (%)	
Diet	5 (55.6)
Oral hypoglycemic agents	2 (22.2)
Insulin	2 (22.2)

Abbreviations: GDM, gestational diabetes mellitus

Concerning motivational text messages, a total of 120 (100%) messages were successfully sent to all the participants (*n* = 15) who had access to the app during the study period, with 2 messages per week per participant. Of these, 49 (68.06%) were acknowledged by the participants. Unfortunately, none of the participants were able to encode their healthy behaviors (e.g., body weight, blood sugar, diet behaviors, and physical activity) into the SEESPA because of a technical issue that prevented them from saving their data.

### Acceptability of the SEESPA

Table 3 showed the users’ reported acceptability of SEESPA, 4 weeks following the utilization of the app. All the nine women who agreed to participate in the post-trial evaluation reported that the app is useful and, overall, they felt satisfied with the application. The majority of the participants accessed the app between two (*n* = 3, 33.3%) and four times (*n* = 3, 33.3%) per week, with one woman used the app at least once per week.

**Table 3:** Users’ Acceptability of the SEESPA

Participant Code	Usefulness (Yes/ No)	Effectiveness (0–10)	Usage per Week	Satisfaction (Satisfied/ Not Satisfied)	Motivators	Barriers and Suggestions for Improvement
1	Yes	5	4	Satisfied	-Excellent information about selection of appropriate diet based in sugar level -Diet and physical activity sections -BS recording section -Text messages	-Do not have the feature to record the previous body weight reading, which isfrom the beginning of pregnancy -Do not have the feature to encode BS data for the missing days
2	Yes	8	1	Satisfied	-Plenty of useful health information is included in the app	-Psychology of the pregnancy and pregnancy-related fatigues and discomforts that prevents women from recording and tracking the healthy behaviors
4	Yes	7	3	Satisfied	-Being a pregnant woman diagnosed with GDM -App has lots of useful new information about GDM, and diet that increases awareness	-Technical issues on the cellphone, which prevented the regular usage
5	Yes	8	2	Satisfied	-GDM information - Text health messages -Role model video	-Do not have feature to enter numbers with decimals when recording BS -Not able to transfer the recorded data of blood glucose from the glucometer to the app
6	Yes	9	3	Satisfied	-Very good information about GDM -Very good organization and easy to use -Diet section and selection of appropriate fruits and dairy based on BS level and fat -PA activity section	- Doctor has no access to the patient’s daily log in the app
7	Yes	8	4	Satisfied	- Useful information about GDM -Friendly and easy to use -Role model video	-Busy with the house responsibility and study work
9	Yes	8	2	Satisfied	-Diet section -Motivational messages	-Was already using another pregnancy application before introducing this application
14	Yes	9	2	Satisfied	-App has lots of good information about GDM -Diet section	-Not able to share daily logs of healthy behaviors to the primary physician
15	Yes	7	4	Satisfied	-The text messages -The health information about GDM, diet, and tips for physical activity -Role model video	-Not able to see changes in the body weight when I update my latest body weight reading - Busy with family responsibilities - Forgot to use - Prior use of other pregnancy apps

Abbreviations: SEESPA, self-efficacy-enhancing smartphone application; BS, blood sugar; GDM, gestational diabetes mellitus; PA, physical activity

Table 3 shows the participants’ rating of the effectiveness of SEESPA in improving women’s self-efficacy for behavioral modifications using a rating scale from 0 to 10. Six out of the nine women rated the effectiveness of the app 8 and above. Further, the majority of the participants appreciated that the app has thorough health information on GDM, tips on diet selection, safe physical activity, and is easy to use, thus providing a novel, comprehensive tool for GDM self-care management. They further appreciated the motivational text messages and role model video as motivational factors to use the app.

Regarding barriers to use the app and suggestions for improvement, two of the participants indicated they were busy with the house and family responsibilities, two stated that their primary physician has no access to the self-reported data, two mentioned because of the pregnancy-related fatigue, and two stated because they were already using other pregnancy smartphone apps. In addition, some of the participants indicated that they faced technical issues with the app such as unable to enter their blood sugar readings with decimal numbers, and unable able to track changes in their body weight (Table 3).

Participants’ suggestions to improve the app were reported in Table 4. The participants suggested to send daily reminders to check blood sugar before or after meals, include complete meal suggestions in the diet section, synchronize the app to glucometer and step-counts apps, introduce the app immediately after GDM diagnosis, give the ability to share the recorded data to the primary physician, and give immediate suggestions to improve blood sugar level if it is too high or too low.

**Table 4:** Users’ Suggestions to Improve SEESPA

Participant Code	Suggestions for Improvement
1	•Add daily reminders for BS recording after each meal •Sync SEESPA with step -count applications for physical activity
2	•None
4	•Include healthy meal suggestions •Provide immediate advice to improve BS level if it is too high or too low
5	•Enable entering numbers with a decimal for the BS readings
6	•Consider daily reminders to record BS readings fasting and after meals
7	•Include healthy meal suggestions
9	•Introduce the application immediately after GDM diagnosis
14	•Consider the ability to share the recorded data with the primary healthcare provider to get a comprehensive picture of self-care activities done
15	•Provide prompt suggestions for the users about the recommended interventions to improve their blood glucose level if it is too high or too low •Include more attractive pictures in the GDM health information section

Abbreviations: SEESPA, self-efficacy-enhancing smartphone application; BS, blood sugar; GDM, gestational diabetes mellitus

## Discussion

This study presents a pilot trial that is designed to examine the feasibility and the acceptability of the SEESPA intervention among pregnant women with gestational diabetes. The development of this app is grounded on the health belief model with more focus on including self-efficacy enhancing strategies. SEESPA incorporates thorough information about gestational diabetes, selection of healthy diet, tips for safe exercises, and regular tracking of blood glucose level, body weight, diet, and physical activity. Overall, SEESPA was experienced as feasible and acceptable, with almost all of the participants (except one participant) used and accessed the app at least for 4 weeks (trial period).

The utilization of the app was found to be feasible, with a 100% recruitment rate of the eligible participants, and 60.0% retention rate of the enrolled participants. Although the retention rate (60.0%) is below the ideal retention rate (80.0%) to indicate high feasibility of the SEESPA (Eldridge, et al., [9]), it is nevertheless an essential finding given that it is challenging to engage pregnant women in lifestyle changing behaviors during a short span like pregnancy (Hill et al., [13]), knowing all the physiological changes (McKinley et al., [19]), and pregnancy-related discomfort that happen during pregnancy.

In our study, we integrated motivational text messages and role model video in the SEESPA to motivate the participants to adhere to the GDM-related self-care management and to modify their lifestyle behaviors. Our results show that the study participants liked the role model video and considered it as a motivating factor to use the app. Further, the majority of the motivational text messages were acknowledged and appreciated by the participants, suggesting the participant’s interest and interaction in using the app to foster their self-care behaviors. In Nicholas et al. study (Nicholas et al., [22]), the participants appreciated the motivational text messages embedded within the START app as it provided them a sense of competence to adhere to self-monitoring. This study finding supports previous mobile health (mHealth) interventions that recommended the use of motivational text messages to promote behavioral changes among general adults (Kinnafick, Thøgersen-Ntoumani, & Duda, [17]; Samdal et al., [27]). It is therefore recommended that these app features (motivational messages and role model video) are considered in future technology-based health promotion interventions aimed at modifying lifestyle behaviors.

With regard to acceptability, telephone interviews with the study participants showed that SEESPA was viewed favorably in terms of the overall satisfaction, usefulness, and effectiveness to improve self-efficacy for adherence to healthy behaviors. This finding is consistent with scientific findings suggesting that the developed smartphone applications were acceptable, useful, and convenient for SMBG level among women with GDM (Jo, & Park, [16]).

On the other hand, the study participants reported some of the barriers that limited their frequent engagement with the SEESPA. First, all of the participants reported the technical issue encountered with the app that prevented them from saving their data, entering numbers with decimals for blood sugar reading, and entering blood sugar readings for the missing days. Consequently, the app designers were informed to investigate the cause and fix the reported technical issue. Second, few participants stated the inconvenience of manually recording their blood sugar level, suggesting synchronizing with the commonly available glucometers. One of the essential features of smartphone apps developed for blood sugar monitoring is the automatic transfer of the blood sugar readings from the glucometer to the smartphone (Borgen et al., [5]). This feature is expected to enhance users’ regular usage of the app and it provides a mean to maintain the accuracy of the blood sugar recordings (Borgen et al., [5]). In the current study, this feature was not considered during the designing of the SEESPA due to the limited financial fund available to support the app development. Further, one participant suggested synchronizing the SEESPA with a step-count smartphone app, to promote the users’ engagement with the physical activity and for the practical use of the app. Thus, the authors will consider embedding these two features (sync SEESPA with a glucometer and step-count apps) in the improved version of SEESPA to make it more appealing and valued by the future users. Third, two participants indicated that SEESPA has no feature to forward the self-reported data to the primary physician. Therefore, a print function of the data will be implemented in the final version of the app to use in the regular check-up of the user with their primary health care providers. Lastly, access to the SEESPA was provided to the participants weeks after their pregnancy and diagnosis with GDM. As the current pregnant women are from younger generations who are interested in using mHealth technologies to monitor their pregnancy, one of the study participants mentioned that the prior use of other pregnancy apps made her reluctant to use the SEESPA. On the other hand, another participant suggested introducing SEESPA immediately after the diagnosis with GDM to enhance adherence to the recommended behaviors in the app.

Self-management of GDM is a vital part of optimizing glycemic control and is the first line of treatment for gestational diabetes. The utilization of smartphone apps demonstrated effectiveness in improving glycemic control and attitude of general adults with diabetes mellitus (Charron-Prochownik et al., [8]; Holmes et al., [15]). Successful implementation and adoption of mHealth based technological interventions, however, requires the intervention to be acceptable by the target users and standardized into practice. Therefore, in the current study, the target users (Arabic-speaking women with GDM) were involved in testing the first version of the SEESPA to increase its wide-scale adoption and dissemination among Arabic-speaking women with GDM. The study participants brought few suggestions to improve users’ engagement in self-care activities and interaction with the SEESPA (Table 4). Based on discussion with the participants and on their suggestions, there is an indication of knowledge deficit and lack of competence for the specific area of glycemic control. Providing prompt advice is an appreciated method of providing targeted and individualized health information (Antypas, & Wangberg, [2]). In our newly developed app, after checking blood sugar, participants received an immediate alert indicating the normality of their blood glucose levels. However, targeted advice was not considered during the development of the app. Therefore, it would be beneficial for future work to consider integrating similar features in any newly developed smartphone apps. Further, the study PI will embed these features in the final version of the SEESPA.

To the best of our knowledge, this is the first pilot trial study that incorporating self-efficacy enhancing strategies in a smartphone app to promote healthy lifestyle behaviors among pregnant women with GDM in an Arabic context. The major strength of this experimental trial is that the SEESPA was grounded on a theoretical framework (HBM and self-efficacy). This characteristic gave greater confidence in the expected positive impact of SEESPA to promote behavioral changes. In addition, SEESPA has been designed using the leading mobile operating systems (iOS and Android), so users of both systems had the chance to participate and test acceptability of the app.

In addition to the above-study strengths, several limitations in the study were acknowledged. Although the integrated features of SEESPA were found acceptable and useful by the participants, we were not able to assess objectively the participants’ app usage for the self-reported data because of the technical issues. We recommend conducting regular follow-up during the trial period with the users to investigate and solve any arising technical issues in future research. Also, the sample size in the end-user testing phase was limited. However, the qualitative data from the telephone interview gave comprehensive insights about the SEESPA from the users’ perspective.

## Conclusion

The prevalence of GDM-related both short- and long-term complications is growing, and improved self-care activities among women with GDM is urgently needed to decrease the incidence of maternal and neonatal complications, particularly in low- and middle-income countries. Self-care through modifications of lifestyle behaviors is a core recommendation of the GDM management. There is growing attention to the use of mHealth technologies to promote glycemic control for women with GDM around the world, but research on promoting change of health behaviors is lacking. A health intervention delivered by smartphone apps could be useful, acceptable, potentially cost-effective, and continuously motivating.

Our objective in the current study was to utilize the opportunity of an essential ‘teachable moment’ and develop an innovative technology-based intervention to promote self-efficacy for behavioral changes to improve pregnancy outcomes of both the mother and her fetus. The study results indicate that the developed innovative SEESPA is both clinically feasible and acceptable to be used by pregnant women with GDM. Following this pilot study (phase 1), we are planning to conduct a randomized clinical trial (phase 2) to compare the effectiveness of the final version of SEESPA with the golden standard of the traditional health education and the standard prenatal care (control) provided for women with GDM. Findings from phase 2 are expected to inform the health policymakers to integrate SEESPA with the antenatal health care practice of women with GDM, specifically in developing countries where there is a greater risk of developing GDM complications among mothers and their infants. This is an essential future strategy to be considered, given the considerable shortfalls in the GDM knowledge and competence for glycemic control among pregnant women with gestational diabetes around the world and in developing countries.

### Funding sources

This work was supported by the Internal Grant [*IG/CON/MCHH/19/02*] at Sultan Qaboos University, Sultanate of Oman. The funder provided financial support, however was not involved in design of the study; collection, analysis, and interpretation of data; or writing the manuscript.

## Supplementary Information

**Additional file 1.**

## Data Availability

All data generated or analyzed during this study are included in this published article and its supplementary information files.

## Abbreviations

GDM: Gestational diabetes mellitus
SEESPA: Self-efficacy enhancing smart-phone application
SMBG: Self- monitoring blood glucose
RCT: Randomized controlled trial
mHealth: Mobile health
HBM: Health belief model
NIDDKD: National Institutes of Diabetes and Digestive and Kidney Diseases
SMOG: Simple Measure of Gobbledygook
BMI: Body mass index**;** PI, principal investigator

## References

[1] Al-Hashmi I, Hodge F, Nandy K, Thomas E, Brecht ML (2018). The effect of a self-efficacy-enhancing intervention on perceived self-efficacy and actual adherence to healthy behaviours among women with gestational diabetes mellitus. Sultan Qaboos Univ Med J.

[2] Antypas K, Wangberg SC (2014). Combining users’ needs with health behavior models in designing an internet-and mobile-based intervention for physical activity in cardiac rehabilitation. JMIR Res Protoc.

[3] Arain M, Campbell MJ, Cooper CL, Lancaster GA (2010). What is a pilot or feasibility study? A review of current practice and editorial policy. BMC Med Res Methodol.

[4] Bellamy L, Casas JP, Hingorani AD, Williams D (2009). Type 2 diabetes mellitus after gestational diabetes: A systematic review and meta-analysis. Lancet.

[5] Borgen I, Garnweidner-Holme LM, Jacobsen AF (2017). Smartphone application for women with gestational diabetes mellitus: a study protocol for a multicenter randomized controlled trial. BMJ Open.

[6] Boulos MNK, Brewer AC, Karimkhani C, Buller DB, Dellavalle RP (2014). Mobile medical and health apps: state of the art, concerns, regulatory control and certification. Online Journal of Public Health Inform.

[7] Carolan M, Steele C, Margetts H (2010). Knowledge of gestational diabetes among a multi-ethnic cohort in Australia. Midwifery.

[8] Charron-Prochownik D, Sereika SM, Becker D (2013). Long-term effects of the booster-enhanced READY-Girls preconception counseling program on intentions and behaviors for family planning in teens with diabetes. Diabetes Care.

[9] Eldridge SM, Chan CL, Campbell MJ (2016). CONSORT 2010 statement: extension to randomised pilot and feasibility trials. BMJ.

[10] Garnweidner-Holme LM, Borgen I, Garitano I, Noll J, Lukasse M (2015). Designing and developing a mobile smartphone application for women with gestational diabetes mellitus followed-up at diabetes outpatient clinics in Norway. Healthcare (Basel).

[11] Gasim T. Gestational diabetes mellitus: Maternal and perinatal outcomes in 220 Saudi women. Oman Med J. 2012;27(2):140. 10.5001/omj.2012.29 PMC3321340 22496940

[12] Gilbert L, Gross J, Lanzi S, Quansah DY, Puder J, Horsch A (2019). How diet, physical activity and psychosocial well-being interact in women with gestational diabetes mellitus: an integrative review. BMC Pregnancy Childbirth.

[13] Hill B, McPhie S, Moran LJ, Harrison P, Huang TK, Teede H, Skouteris H (2017). Lifestyle intervention to prevent obesity during pregnancy: Implications and recommendations for research and implementation. Midwifery.

[14] Hirst JE, Mackillop L, Loerup L (2015). Acceptability and user satisfaction of a smartphone-based, interactive blood glucose management system in women with gestational diabetes mellitus. J Diabetes Sci Technol.

[15] Holmes VA, Spence M, McCance DR, Patterson CC, Harper R, Alderdice FA (2012). Evaluation of a DVD for women with diabetes: impact on knowledge and attitudes to preconception care. Diabet Med.

[16] Jo S, Park HA (2016). Development and evaluation of a smartphone application for managing gestational diabetes mellitus. Healthc Inform Res.

[17] Kinnafick FE, Thøgersen-Ntoumani C, Duda J (2016). The effect of need supportive text messages on motivation and physical activity behaviour. J Behav Med.

[18] Macaulay S, Dunger DB, Norris SA. Gestational diabetes mellitus in Africa: A systematic review. PLoS ONE. 2014;9(6):e97871. 10.1371/journal.pone.0097871. 10.1371/journal.pone.0097871 PMC4043667 24892280

[19] McKinley MC, Allen-Walker V, McGirr C, Rooney C, Woodside JV (2018). Weight loss after pregnancy: Challenges and opportunities. Nutr Res Rev.

[20] National Institute of Diabetes and Digestive and Kidney Diseases. Gestational diabetes. 2017. Accessed January 2017. https://www.niddk.nih.gov/health-information/diabetes/overview/what-is-diabetes/gestational/definition-facts

[21] National Institutes of Diabetes and Digestive and Kidney Diseases (NIDDKD). Gestational diabetes. 2017. Retrived from: https://www.niddk.nih.gov/health-information/diabetes/overview/what-is-diabetes/gestational.

[22] Nicholas JC, Ntoumanis N, Smith BJ, Quested E, Stamatakis E, Thøgersen-Ntoumani C (2021). Development and feasibility of a mobile phone application designed to support physically inactive employees to increase walking. BMC Med Inform Decis Mak.

[23] Nwolise CH, Carey N, Shawe J (2017). Exploring the acceptability and feasibility of a preconception and diabetes information app for women with pregestational diabetes: A mixed-methods study protocol. Digital Health.

[24] Pellegrini CA, Pfammatter AF, Conroy DE, Spring B (2015). Smartphone applications to support weight loss: current perspectives. Advanced Health Care Technologies.

[25] Plows JF, Stanley JL, Baker PN, Reynolds CM, Vickers MH (2018). The pathophysiology of gestational diabetes mellitus. Int J Mol Sci.

[26] Rosenstock I, Strecher V, Becker M (1988). Social learning theory and the health belief model. Health Education Quarterly.

[27] Samdal GB, Eide GE, Barth T, Williams G, Meland E (2017). Effective behaviour change techniques for physical activity and healthy eating in overweight and obese adults; systematic review and meta-regression analyses. Int J Behav Nutr Phys Act.

[28] Thomas LW (1995). A critical feminist perspective of the health belief model: Implications for nursing theory, research, practice and education. J Prof Nurs.

[29] Turki G (2012). Gestational diabetes mellitus: Maternal and perinatal outcomes in 220 Saudi women. Oman Med J.

[30] World Health Organization. Working with individuals, families and communities to improve maternal and newborn health. 2003. Accessed January 2017. https://apps.who.int/iris/bitstream/handle/10665/83795/WHO_FCH_RHR_03.11.pdf?sequence=1&isAllowed=y.

