# Supplementary material for: Development, feasibility and acceptability of a self-efficacy-enhancing smartphone application among pregnant women with gestational diabetes mellitus: single- arm pilot clinical trial
Source: BMC Pregnancy Childbirth. 2022 Apr 23;22:358. doi: 10.1186/s12884-022-04684-1 (PMC9034265; doi:10.1186/s12884-022-04684-1)
Supplement: Supplementary file 1 — Additional file 1. [file 12884_2022_4684_MOESM1_ESM.docx]

SEESPA Results

| Participant No. | Demographic | Phone | Useful  Yes/no | Effectiveness  0-10 | Satisfaction | Motivators | Barriers | Suggestions for Improvement |
| --- | --- | --- | --- | --- | --- | --- | --- | --- |
| 1 | Age: 36  BMI: 24  Treatment: Insulin | Android | Yes | 5 | Satisfied (excellent) | Excellent information about selection of appropriate diet based in sugar level.  Diet and physical activity sections.  BS recording section.  Text messages. | Do not have the feature to record the previous body weight reading, which isfrom the beginning of pregnancy.  Do not have the feature to encode BS data for the missing days. | Add daily reminders for BS recording after each meal.  Sync SEESPA with step -count applications for physical activity. |
| 2 | Age: 32  BMI: 21  Treatment: diet | Android | Yes | 8 | Satisfied (excellent) | Plenty of useful health information is included in the app. | Psychology of the pregnancy and pregnancy-related fatigues and discomforts that prevents women from recording and tracking the healthy behaviors. | None |
| 4 | Age: 29  BMI: 25  Treatment: diet | ios | Yes | 7 | Satisfied (very good) | Being a pregnant woman diagnosed with GDM.  App has lots of useful new information about GDM, and diet that increases awareness. | Technical issues on the cellphone, which prevented the regular usage. | Include healthy meal suggestions.  Provide immediate advice to improve BS level if it is too high or too low. |
| 5 | Age: 25  BMI: 18.5  Treatment: diet | ios | Yes | 8 | Satisfied (very good) | GDM information  Text health messages  Role model video | Do not have feature to enter numbers with decimals when recording BS.  -Not able to transfer the recorded data of blood glucose from the glucometer to the app. | Enable entering numbers with a decimal for the BS readings. |
| 6 | Age: 26  BMI: 29  Treatment: Oral tab | ios | Yes | 9 | Satisfied (Excellent ) | Very good information about GDM.  Very good organization and easy to use.  Diet section and selection of appropriate fruits and dairy based on BS level and fat.  PA activity section. | Doctor has no access to the patient’s daily log in the app. | Consider daily reminders to record BS readings fasting and after meals. |
| 7 | Age: 28  BMI: 23  Treatment: diet | ios | Yes | 8 | Satisfied (Excellent) | Useful information about GDM.  Friendly and easy to use.  Role model video. | Busy with the house responsibility and study work. | Include healthy meal suggestions. |
| 9 | Age: 30  BMI: 23  Treatment: Oral tab | Ios | Yes | 8 | Satisfied (Excellent) | Diet section.  Motivational messages. | Was already using another pregnancy application before introducing this application. | Introduce the application immediately after GDM diagnosis. |
| 14 | Age: 37  BMI: 25  Treatment: insulin | ios | Yes | 9 | Satisfied (Excellent) | App has lots of good information about GDM.  Diet section. | Not able to share daily logs of healthy behaviors to the primary physician. | Consider the ability to share the recorded data with the primary healthcare provider to get a comprehensive picture of self-care activities done. |
| 15 | Age: 32  BMI: 22  Treatment: diet | Ios | Yes | 7 | Satisfied (Very good) | The text messages.  The health information about GDM, diet, and tips for physical activity.  -Role model video. | Not able to see changes in the body weight when I update my latest body weight reading.  Busy with family responsibilities.  Forgot to use.  Prior use of other pregnancy apps. | Provide prompt suggestions for the users about the recommended interventions to improve their blood glucose level if it is too high or too low.  Include more attractive pictures in the GDM health information section. |
| 2 | Age: 33  BMI: 20  Treatment: diet |  |  |  |  |  |  |  |
| 3 | Age: 35  BMI: 23.5  Treatment: Oral tab |  |  |  |  |  |  |  |
| 8 | Age: 27  BMI: 21  Treatment: diet |  |  |  |  |  |  |  |
| 10 | Age: 28  BMI: 24  Treatment: diet |  |  |  |  |  |  |  |
| 11 | Age: 36  BMI: 25  Treatment: Insulin |  |  |  |  |  |  |  |
| 12 | Age: 30  BMI: 21  Treatment: diet |  |  |  |  |  |  |  |
| 13 | Age: 30  BMI: 29  Treatment: Oral tab |  |  |  |  |  |  |  |

| Participants characteristics |  |  |  |
| --- | --- | --- | --- |
|  |  |  |  |
|  |  |  |  |
|  |  |  |  |
|  |  |  |  |
|  |  |  |  |
|  |  |  |  |
|  |  |  |  |
|  |  |  |  |
|  |  |  |  |
